# Proximity to overhead power lines and childhood leukaemia: an international pooled analysis

**DOI:** 10.1038/s41416-018-0097-7

**Published:** 2018-05-29

**Authors:** Aryana T Amoon, Catherine M Crespi, Anders Ahlbom, Megha Bhatnagar, Isabelle Bray, Kathryn J Bunch, Jacqueline Clavel, Maria Feychting, Denis Hémon, Christoffer Johansen, Christian Kreis, Carlotta Malagoli, Fabienne Marquant, Camilla Pedersen, Ole Raaschou-Nielsen, Martin Röösli, Ben D Spycher, Madhuri Sudan, John Swanson, Andrea Tittarelli, Deirdre M Tuck, Tore Tynes, Ximena Vergara, Marco Vinceti, Victor Wünsch-Filho, Leeka Kheifets

**Affiliations:** 1 Department of Epidemiology, University of California Los Angeles Fielding School of Public Health, Los Angeles, CA 90095-1772 USA; 2 Department of Biostatistics, University of California Los Angeles Fielding School of Public Health, Los Angeles, CA 90095-1772 USA; 3 Unit of Epidemiology, Institute of Environmental Medicine, Karolinska Institutet, Stockholm, Sweden; 4 Department of Health and Social Sciences, University of the West of England, Bristol, BS16 1QY UK; 5 National Perinatal Epidemiology Unit, Nuffield Department of Population Health, University of Oxford, Headington, Oxford, OX3 7LF UK; 6 Epidemiology of Childhood and Adolescent Cancers, CRESS, INSERM, UMR 1153, Paris Descartes University, Villejuif, France; 7 National Registry of Childhood Cancers - Hematological Malignancies, Villejuif, France; 8 The Danish Cancer Society Research Center, Strandboulevarden 49, 2100 Copenhagen, Denmark; 9 Oncology Clinic, Finsen Center, Rigshospitalet 5073, 2100 Copenhagen, Denmark; 10 Institute of Social and Preventive Medicine (ISPM), University of Bern, Bern, Switzerland; 11 Research Center of Environmental (CREAGEN), Genetic and Nutritional Epidemiology University of Modena and Reggio Emilia, Modena, Italy; 12 Department of Environmental Science, Aarhus University, Frederiksborgvej 399, 4000 Roskilde, Denmark; 13 Department of Epidemiology and Public Health, Swiss Tropical and Public Health Institute, Basel, Switzerland; 14 University of Basel, Petersgraben 1, Basel, Switzerland; 15 Department of Public Health, Aarhus University, Aarhus, Denmark; 16 College of Osteopathic Medicine of the Pacific, Western University of Health Sciences, Pomona, CA 91766-1854 USA; 17 National Grid, London, UK; 18 Cancer Registry Unit, National Cancer Institute, Milan, 20133 Italy; 19 School of Medicine, University of Tasmania, Hobart, TAS Australia; 20 Royal Hobart Hospital, Hobart, TAS Australia; 21 Department of Occupational Health Surveillance, National Institute of Occupational Health, Oslo, Norway; 22 Energy and Environment Sector, Electric Power Research Institute, Palo Alto, CA 94304 USA; 23 Department of Epidemiology, Boston University School of Public Health, Boston, MA USA; 24 Department of Epidemiology, School of Public Health, University of São Paulo, São Paulo, 01246-904 Brazil

**Keywords:** Leukaemia, Cancer epidemiology, Paediatric cancer, Leukaemia, Cancer epidemiology

## Abstract

**Background:**

Although studies have consistently found an association between childhood leukaemia risk and magnetic fields, the associations between childhood leukaemia and distance to overhead power lines have been inconsistent. We pooled data from multiple studies to assess the association with distance and evaluate whether it is due to magnetic fields or other factors associated with distance from lines.

**Methods:**

We present a pooled analysis combining individual-level data (29,049 cases and 68,231 controls) from 11 record-based studies.

**Results:**

There was no material association between childhood leukaemia and distance to nearest overhead power line of any voltage. Among children living < 50 m from 200 + kV power lines, the adjusted odds ratio for childhood leukaemia was 1.33 (95% CI: 0.92–1.93). The odds ratio was higher among children diagnosed before age 5 years. There was no association with calculated magnetic fields. Odds ratios remained unchanged with adjustment for potential confounders.

**Conclusions:**

In this first comprehensive pooled analysis of childhood leukaemia and distance to power lines, we found a small and imprecise risk for residences < 50 m of 200 + kV lines that was not explained by high magnetic fields. Reasons for the increased risk, found in this and many other studies, remains to be elucidated.

## Introduction

Thirty-five epidemiologic studies have examined the association between exposure to extremely low-frequency magnetic fields (MFs) and childhood leukaemia^1^. Analyses that have pooled data from multiple studies^2–5^ report a small but consistent increased risk of childhood leukaemia associated with exposures above 0.3 or 0.4 μT. In one of these analyses, Kheifets et al.^4^ pooled six studies for an analysis of the association between distance from power lines and childhood leukaemia. They found an odds ratio (OR) of 1.59 (95% confidence interval (CI): 1.02–2.50) for the closest distance category, which was comparable to the result for MF. High MF can occur close (e.g., < 100 m) to high voltage power lines^6^. However, distance is known to be a poor predictor of MF exposure^7^ and therefore the question arises as to whether the association of increased childhood leukaemia risk with distance is due to MF or to other factors associated with distance from overhead power lines that are unrelated to long-term average MF. Unlike MF, there has not yet been a comprehensive pooled analysis on childhood leukaemia and distance to power lines, which could help to answer this question.

Draper et al.^8^, reporting on a study in the United Kingdom (UK) using diagnosed cases from 1962–1995, found an association between childhood leukaemia and the distance between home address at birth and the nearest high-voltage overhead line^8^ with the apparent risk extending out to 600 m, a distance greater than would be expected for MF from high-voltage lines, because MF rapidly decline with distances and are very weak at distances beyond 100 m^9,10^. Whether the risk truly persists at greater distances from power lines and what might be an explanation for this observation is unclear. Several explanations have been proposed, including selection of controls, but none are fully satisfactory^11^, leaving open the possibility that some factor associated with distance other than MF is responsible.

The study by Draper et al.^8^ was extended to cover more recent time periods (diagnoses during 1962–2008) and lower line voltages^12^. The updated study confirmed the raised leukaemia risks reported for the earlier decades, but found that risk declined in the latest decades. A small Danish study of calculated fields also found higher risks in earlier decades (1968–1986) compared with more recent cases (1987–2003)^13^. Two large studies in France and the United States, specifically California, reported that living within 50 m of a 200 + kV line may be associated with a small increased risk of childhood leukaemia^14,15^. In these studies, no increase in risk was observed beyond 50 m from 200 + kV lines or within 50 m of lower voltage lines. Both studies covered more recent time periods only (diagnosed in 1988 or later). Thus, the existence of similar temporal trends in risk in other countries is unresolved.

Geographic information systems, maps and on-site measurements have all been used to assess proximity to power lines^16^, each with varying degrees of accuracy. In addition, the point of the home chosen for the start of measurement of the distance varied from study to study; some used the centre of the building^17^, whereas others used the corner closest to the power line^18,19^ or where the mailbox was located^14^. Some studies identified observations with poor geocoding accuracy and excluded them from analyses. If the association were real, one would expect it to be stronger when data with problematic geocoding are excluded from the analysis. On the other hand, such exclusions might inadvertently introduce bias.

In Sweden^18^, the MF association with childhood leukaemia was limited to single-family homes, although calculated MF levels were somewhat higher in apartments mainly due to fields from sources other than power lines, as verified by spot measurements. This resulted in lower correlation between calculated fields and spot measurements for apartments compared with single-family homes, which may explain why the association between calculated fields and childhood leukaemia was limited to homes with better exposure prediction (i.e., single-family homes).

The association between socioeconomic status (SES) and leukaemia is complex and varies based on the specific measures used. Individual measures such as high family income tend to be associated with a lower risk of childhood leukaemia in most studies, whereas the opposite is true for ecological measures such as percent of neighbourhood unemployment or deprivation index^20–24^. Study participants often differ in SES and other factors from non-participants, possibly resulting in selection bias^25–27^, but this is less of an issue in the record-based studies that comprise this analysis, which do not require active participation. Indeed, Poole et al.^22^ argues that individual measures of SES often come from case–control studies requiring participation, whereas ecological measures often come from record-based studies less prone to this bias. In addition, residence in single-family homes may be associated with higher SES and with various exposures (including both distance and MFs) and thus potentially confound an association.

Power lines may be co-located with other potential risk factors such as motorways or railways, resulting in higher traffic-related air pollution exposure in proximity to power lines^28,29^ or specifically higher nitrogen dioxide exposure from traffic^30^. Several studies have reported associations between childhood leukaemia and traffic density, proximity to major roads or highways, or exposure to air pollutants caused by traffic. A meta-analysis by Boothe et al.^31^ assessing childhood leukaemia in relation to multiple pollutants found an increased risk for post-natal exposure but no association with pre-natal exposure. Most studies found an association with childhood leukaemia overall, but the association tended to be stronger when examining just acute lymphoblastic leukaemia (ALL) or acute myeloid leukaemia (AML) for specific pollutants^32^.

Studies of childhood leukaemia and distance from power lines have assessed exposure at the birth home and/or diagnosis home. The critical time-period of exposure for a potential effect on leukaemia development is unknown and it is unclear whether birth home or diagnosis home is more representative of a child’s lifetime exposure, and/or which exposure period is more relevant biologically. Of course, the former depends on the pattern of movement of the family between pregnancy and diagnosis. Residential mobility can manifest as selection bias, confounding or increased measurement error, or it could also be a potential risk factor^33^.

There are many unresolved issues regarding the association between childhood leukaemia risk and distance from overhead power lines that are difficult to resolve in any single study. In this study, we pool data from multiple studies to provide a more comprehensive assessment of the association between childhood leukaemia risk and distance to power lines than previously attempted. We also assess whether the association is due to MF or other factors, and further consider whether bias, confounding or other methodologic challenges inherent in these studies have substantial influence on the results using available data.

## Methods

### Search and inclusion

The present study is a pooled analysis combining raw individual-level data from multiple studies, sometimes called an individual participant data (IPD) meta-analysis^34,35^. We searched the published literature through PubMed and a database of MF literature (EMF Portal https://www.emf-portal.org/en) to identify studies on childhood leukaemia and proximity to overhead transmission lines. To locate studies potentially missed in our initial searches, we also searched the reference lists in identified papers and conducted an informal survey of epidemiologists involved in MF research. To be included in our analysis, a study must have used record-based exposure assessment, i.e., not requiring active participation of study subjects, with exposure (i.e., distance to power lines) determined at the individual level; thus, studies with ecologic or area-based exposure assessment were excluded. We excluded wire code studies^36–43^. Although wire code studies use distance, they document only the power lines closest to the home, and thus higher voltage power lines might not have been recorded if there were any distribution lines that were closer. Studies with hospital controls were also excluded, because such controls may not be representative of the source population from which cases arose. We identified 21 studies on distance to power lines published between 1993 and 2016, of which 13 met our inclusion criteria (Table 1)^14,15,17–19,44–51^. Eight studies were excluded; reasons for their exclusion are provided in the appendix (Table S1)^52–59^.

**Table 1:** Characteristics of studies meeting criteria for pooled childhood leukaemia and distance to power lines

Country (author, year)	Population (leukaemia)	Years of diagnosis	Age	Voltages (kV)	Home analysed	Homes with data Diagnosis (cases only)	Results (shortest distance category to reference)	Adjusted for
Included:	Cases/controls							
Brazil (Wünsch-Filho et al., 2011)^44^	162/565	2001–2009	0–8	88, 138, 230, 345, 440, 750	Diagnosis	Birth	COR (95% CI): 0.68 (0.25–1.84)	Age, sex, race, mobility, education, day care, Down’s syndrome, flu history, maternal age, maternal occupational history, maternal smoking and alcohol history
						Diagnosis	AOR (95% CI): 1.54 (0.26–9.12)	
Denmark (Pedersen et al., 2014)^45^	1698/3396	1968–2006	0–15	132, 150, 220, 400	Birth	Birth	COR (95% CI): 0.76 (0.40–1.45)	Socioeconomic status
							AOR (95% CI): 0.76 (0.40–1.45)	
France (Sermage-Faure et al., 2013)^14^	2712/29797	2002–2007	0–14	63, 90, 150, 225, 400	Diagnosis	Diagnosis	AOR (95% CI): 1.2 (0.8–1.9)	Age, departement
Italy 1 (Bianchi et al., 2000)^46^	119/476	1978–1997	0–14	132, 220, 380	Diagnosis	Birth	NA—distance not assessed in publication	Age, sex
						Diagnosis		
Italy 2 (Malagoli et al., 2010)^47^	46/184	1986–2007	0–14	132, 380	Exposed^a^	Birth	NA—distance not assessed in publication	Age, sex, paternal and maternal education, paternal income
						Diagnosis		
Norway (Tynes and Haldorsen, 1997)^19^	148/579	1965–1989	0–14	11, 18, 22, 24, 50, 60, 66, 132, 300, 420	Exposed^a^	Birth	COR (95% CI): 0.6 (0.3–1.3)	
						Diagnosis		
Sweden (Feychting anf Alhbom, 1993)^18^	39/151	1960–1985	0–16	20, 50, 70, 130, 220, 400	Diagnosis	Birth	COR (95% CI): 2.9 (1.0–7.3)	
						Diagnosis		
Switzerland (Spycher et al., 2011)^[b 48^	1109/5545	1985–2014	0–15	100, 150, 220, 380	NA	Birth	NA—distance not assessed in publication	
						Diagnosis		
Tasmania (Lowenthal et al., 2007)^49^	47/47	1972–1980	0–17	88, 110, 220	All	Birth	NA—only adults assessed in publication	
						Diagnosis		
United Kingdom (Bunch et al., 2014)^50^	17299/21059	1962–2008	0–14	132, 275, 400	Birth	Birth	COR (95% CI): 1.00 (0.75–1.34)	
United States (Crespi et al., 2016)^15^	4879/4835	1988–2008	0–15	60, 69, 70, 115, 138, 230, 288, 500	Birth	Birth	AOR (95% CI): 1.4 (0.7–2.7)	Age, sex, race/ethnicity, socioeconomic status
Not included:								**Reason for non-inclusion**
Finland (Verkasalo et al., 1993)^17^	Total = 134,800	1970–1989	0–19		Exposed		OR (95% CI): 1.47 (0.33–6.60)^c^	Original data not found
Japan (Kabuto et al., 2006)^51^	312/603	1999–2001	0–15		Diagnosis		OR (95% CI): 3.06 (1.31–7.13)	Data not received

*AOR* adjusted odds ratio, *CI* confidence interval, *COR* crude odds ratio, *NA* not applicable, *OR* odds ratio.

^a^Home in region with power lines.

^b^Distance used as confounder.

^c^Odds ratio for a subset of participants obtained from private communication

Table 1 provides a list of the 13 studies meeting our inclusion criteria along with each study’s characteristics and main results. We attempted to obtain data for all 13 studies; however, original individual data on distance for Finland and Japan were unavailable. The 11 included studies were conducted in 10 different countries: Brazil, Denmark, France, Italy (two studies in separate regions), Norway, Sweden, Switzerland, Tasmania, the United Kingdom and the United States (California). Exposure assessment in Brazil involved interviews with mothers as well as direct MF measurements inside the homes of children. However, the distance data used in our study were calculated using only grid maps for the Metropolitan Region of São Paulo without requiring participant involvement^44^.

### Material

Among the three largest studies, accounting for 88% of all cases and 76% of cases closest to lines, two (United Kingdom and United States) were based on birth residencies and one (France) on the residence at time of diagnosis; most of the other studies focused on the residence at time of diagnosis in their original publications, but nearly all had some information available on birth homes as well. To focus on populations with higher exposure prevalence, some studies (Norway and Sweden) captured data from the time the child entered an area defined as homes within specified distances to overhead power lines. For Italy 2, we received data for 1998–2013 for the Modena and Reggio Emilia provinces, which is a broader time period than in their original publication^47^. All studies provided information on sex, age and SES (with the exception of France with no information on sex for controls), five studies provided information on mobility (whether subjects moved between birth and diagnosis dates) and four studies provided data on type of dwelling and traffic exposure. We collected available MF information to examine potential impact from adjustments for calculated fields on distance. Most studies provided calculated MF (Brazil provided measured fields), whereas France, Switzerland and Tasmania had no measured or calculated fields available.

All variables were recoded to make them as compatible as possible. Distance to power lines was coded into four categories as the primary analysis (< 50 m, 50 to < 150 m, 150 to < 300 m, and ≥ 300 m as the reference); these cut points were selected based on available data and previous literature.

The primary analyses estimated risk of any type of childhood leukaemia associated with distance of residence from power lines and was restricted to participants who had study-defined accurate geocoding. A mixture of birth and diagnosis homes was used, based on available data, with the home used in prior publications given preference. We estimated risk for distance from closest overhead power line of any voltage and from closest power line with voltage of ≥ 200 kV. Analyses were adjusted for age at diagnosis, sex (except for France where a dummy variable was used) and SES (either individual or ecological, depending on availability), all of which were coded as categorical variables.

### Statistical analysis

We used two statistical approaches: one-stage meta-analysis and two-stage meta-analysis^60^. In the one-stage approach, a traditional pooled analysis, data from all studies were entered simultaneously into a single mixed-effects logistic regression model with random intercepts for study. In the two-stage approach, effect estimates (log ORs) were obtained for each study separately and then combined using a random-effects meta-analysis model. A sensitivity analysis using the two-stage approach included Japan and Finland for which only summary data were available. The risk estimate for Finland comes from unpublished data from a previous pooled analysis^2^ and provided estimates based on living < 50 m to any voltage line. For the primary analyses, estimates from these two methods were compared. For all further analyses, we used the one-stage approach.

Additional subgroup, confounder and sensitivity analyses were performed. We fitted models for various subgroups: comparing subtypes of leukaemia (ALL and AML), excluding children with Down syndrome, and comparing subjects younger than 5 years with those who were 5 years or older at diagnosis. To evaluate whether the strength of the association changed over time, we stratified by decade of diagnosis in a manner similar to that of Bunch et al.^50^, except that due to small numbers, we grouped the decades as 1960–1980, 1980–2000, and 2000 and later. The latter analysis was conducted both with and without the UK study, because it was the hypothesis generating study.

We examined the effects of confounder adjustments on risk estimates. Confounders examined included residential mobility (moving between the time of birth and diagnosis) for five studies, type of dwelling (single-family home or other) for four studies, traffic exposure (high, medium or low) for four studies, urban vs. rural setting for seven studies, ecological measures of SES for six studies, individual measures of SES for five studies and MF for eight studies. The latter analysis was performed both with and without Brazil, the only country with measured rather than calculated fields. Completeness of collected confounder information varied across studies; many studies with confounder information had substantial subject-level missing data. We further analysed the association between childhood leukaemia risk adjusting for each confounder individually, controlling for age, sex and SES. As confounder information was available only for subsets of studies, we present ORs from both minimally adjusted models (adjusted for age, sex and SES) and models with confounders fit to the same subset of data.

Sensitivity analyses included comparing the association based on birth homes with that in diagnosis homes, as well as the choice of the reference category (e.g., ≥ 300 m vs. ≥ 600 m). To assess how geocoding accuracy may result in exposure misclassification, we conducted an analysis of all observations, regardless of geocoding quality, compared with one including only observations with good geocoding. Finally, we repeated the primary analysis using alternative controls. These analyses used data from studies that assessed other cancers in addition to leukaemia (Italy 2, Sweden, Switzerland, Tasmania, United Kingdom and the United States). We used controls matched to cases of other cancers (central nervous system tumours, lymphoma and other cancers) and conducted an analysis combining all alternative controls.

Analyses were conducted using SAS 9.3 and Stata 14.2.

## Results

**Fig. 1:**
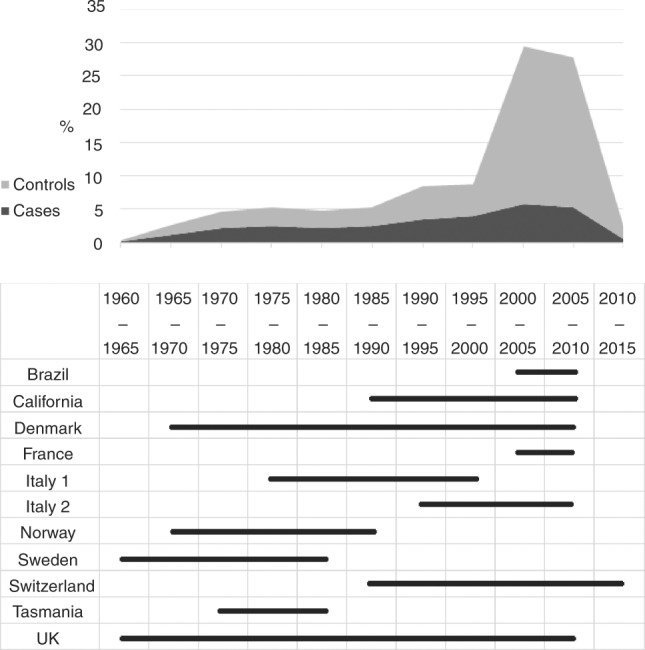
Distribution of cases and controls, and studies by years of diagnosis

Our pooled data set included 30,200 childhood leukaemia cases and 69,594 controls. After restriction to participants with study-defined accurate geocoded distances from overhead power lines to the home, we were left with 97,280 participants (29,049 cases and 68,231 controls). After removing observations with missing data on age, sex or SES, there were 27,143 cases and 65,265 controls available for the primary analysis. Studies included cases diagnosed as early as 1960s and as late as 2014; a larger percentage of cases and controls came from the time periods between 2000 and 2015, as shown in Fig. 1.

Table 2 provides results for the primary analysis using the one-stage approach. There was no material association between childhood leukaemia and distance to nearest line of any voltage for any distance category. Crude ORs and ORs adjusted for age, sex and SES were virtually the same. Results were similar when distance of ≥ 600 m was used as reference (data not shown). For distance to high-voltage lines (200 + kV), there was no difference between risk estimates for distances of 50 to < 150 and 150 to < 300 m compared with those living ≥ 300 m away. However, among those living < 50 m to a 200 + kV power line, the adjusted pooled OR was 1.33 (95% CI: 0.92–1.93). Figure S1 shows the distribution over time of subjects living within 50 m of an overhead power line.

**Table 2:** Odds ratios for childhood leukaemia by distance to closest overhead power lines: one-stage results

Distance (m)	Cases/controls	Crude OR (95% CI)	Adjusted OR (95% CI)
To any voltage			
300 +	25,713/60,603	1.00 (Reference)	1.00 (Reference)
150 to < 300	783/2,559	0.98 (0.89–1.07)	0.98 (0.89–1.07)
50 to < 150	449/1,498	0.98 (0.87–1.10)	0.98 (0.87–1.10)
< 50	198/605	1.02 (0.85–1.21)	1.01 (0.85–1.21)
To 200 + kV line			
300 +	26,434/63,197	1.00 (Reference)	1.00 (Reference)
150 to < 300	304/898	0.97 (0.84–1.12)	0.97 (0.84–1.12)
50 to < 150	152/469	0.98 (0.80–1.20)	0.97 (0.79–1.19)
< 50	50/123	1.35 (0.93–1.94)	1.33 (0.92–1.93)

*CI* confidence interval, *OR* odds ratio, *SES* socioeconomical status.

Analyses were conducted using a random intercept logistic regression model adjusted for age, sex and SES

**Fig. 2:**
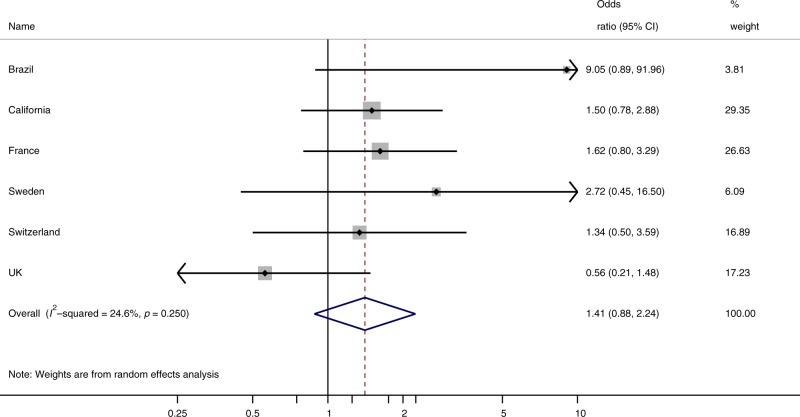
Two-stage meta-analysis < 50 m vs. 300 + m to 200 + kV line*. Values extending beyond the axis are indicated by arrows

Table 3 provides study-specific results and estimates from random effects meta-analysis model based on the two-stage approach. Although the ORs for individual studies for distances < 50 m to a 200 + kV power line ranged from 0.56 (United Kingdom) to 9.05 (Brazil), the results were sufficiently homogenous for pooling: *I*^2^ 24.6%, *p* = 0.25 (Fig. 2). Several smaller studies did not have observations in the < 50 m to a 200 + kV line category (Table 3). The inclusion of estimates from Japan and Finland, for which individual data could not be obtained, only slightly increased the meta-analysis OR. Reassuringly, results of one-stage and two-stage analysis approaches were similar. All further results examine distance to 200 + kV lines and ≥ 300 m as the reference utilising one-stage analysis.

**Table 3:** One-stage and two-stage results for childhood leukaemia comparing < 50 m with 300 + m distance with closest overhead power line

	Any voltage		200 + kV	
Study	Ca/Co	OR (95% CI)	Ca/Co	OR (95% CI)
Included				
Brazil	5/11	1.64 (0.54–4.95)	3/1	9.05 (0.89–91.90)
Denmark	0/2	–	0/0	–
France	23/213	1.17 (0.75–1.81)	9/60	1.62 (0.80–3.30)
Italy1	2/2	4.27 (0.57–31.91)	0/0	–
Italy2	1/4	1.00 (0.10–9.63)	0/0	–
Norway	8/43	0.70 (0.31–1.56)	0/6	–
Sweden	4/8	2.72 (0.45–16.57)	4/8	2.72 (0.45–16.57)
Switzerland	34/199	0.88 (0.61–1.28)	5/20	1.34 (0.50–3.59)
Tasmania	1/0	–	0/0	–
United Kingdom	22/34	0.82 (0.49–1.40)	6/13	0.56 (0.21–1.47)
United States	97/89	1.07 (0.80–1.43)	23/15	1.50 (0.78–2.88)
Two-stage (meta-analysis)		1.02 (0.85–1.22)		1.41 (0.88–2.24)
One-stage (pooled analysis)		1.01 (0.85–1.21)		1.33 (0.92–1.93)
Not included				
Japan		3.06 (1.31–7.13)		–
Finland		1.47 (0.33–6.57)		–
Meta-analysis of all studies		1.10 (0.88–1.38)		–

*Ca* cases, *CI* confidence interval, *Co* controls, *OR* odds ratio, *SES* socioeconoic status.

Denmark and Tasmania had no observations in < 50 m category for any voltage. Italy1, Italy2 and Norway had no observations in the < 50 m category for 200 + kV.

Analyses were adjusted for age, sex (where available) and SES.

Numbers can differ slightly from original publication due to different exclusion criteria

An influence analysis showed that removal of studies one at a time had little effect on the pooled estimate, except that the OR increased from 1.33 to 1.58 on removal of the UK study (Figure S2). The UK study contributed the largest number of participants to the pooled analysis, accounting for over 60% of the cases overall, but only 6 cases and 13 controls lived within 50 m of a 200 + kV line.

### Subgroup analyses

When the analysis was restricted to ALL, the results were similar to those found for the primary analysis, with an OR of 1.39 (95% CI: 0.92–2.10) for children living < 50 m from a 200 + kV power line compared with those ≥ 300 m away (Table 3). The association was not seen for AML (OR: 0.82; 95% CI: 0.27–2.45). Excluding children with Down syndrome had no effect on the results (data not shown).

In the analysis stratified by age at diagnosis, the association between childhood leukaemia and distance < 50 m compared with ≥ 300 m from a 200 + kV line appeared to increase for children diagnosed before age 5 years (OR: 1.65; 95% CI: 1.02–2.67) (Table 4). When examining differences by time period of diagnosis, we found the highest ORs for the years 1960–1980 for all distance categories, followed by the 2000–2010 in the < 50 m category, with virtually null association in the middle decades 1980–2000 (Table 4). When the UK study, which generated the hypothesis of a temporal trend, was excluded from this analysis, ORs were elevated for all time periods in the < 50 m category. However, they were imprecisely estimated, with no apparent trend, and the 1960–1980 period was based on small numbers (Table S2).

**Table 4:** Odds ratios for childhood leukaemia by distance to closest overhead power line of 200 kV or higher within subgroups

Subgroup	Cases	Controls	OR	95% CI
Leukaemia subtype^a^				
Acute lymphoblastic leukaemia				
Distance (m)				
≥ 300	21,068	56,450	1	–
150 to < 300	240	785	0.99	0.84–1.17
50 to < 150	120	418	0.96	0.77–1.21
< 50	40	108	1.39	0.92–2.10
Acute myeloid leukaemia				
Distance (m)				
≥ 300	3,916	33,986	1	–
150 to < 300	48	484	1.02	0.71–1.48
50 to < 150	18	251	0.91	0.50–1.63
< 50	5	68	0.82	0.27–2.45
Age at diagnosis				
< 5 Years				
Distance (m)				
≥ 300	14,940	29,322	1	–
150 to < 300	188	396	1.14	0.94–1.38
50 to < 150	88	228	0.9	0.69–1.17
< 50	34	49	1.65	1.02–2.67
≥ 5 Years				
Distance (m)				
≥ 300	11,683	34,418	1	–
150 to < 300	115	502	0.78	0.62–0.98
50 to < 150	64	241	1.09	0.80–1.49
< 50	16	74	1.01	0.55–1.83
Year of diagnosis				
1960–1980				
Distance (m)				
≥ 300	5,213	5,933	1	–
150 to < 300	40	62	1.71	1.03–2.83
50 to < 150	23	32	2.68	1.34–5.37
< 50	8	12	2.22	0.78–6.33
1980–2000				
Distance (m)				
≥ 300	11,200	13,992	1	–
150 to < 300	110	176	0.89	0.69–1.15
50 to < 150	65	99	1.04	0.75–1.45
< 50	14	22	1.07	0.52–2.18
2000–2010				
Distance (m)				
≥ 300	10,210	43,815	1	–
150 to <300	153	660	0.99	0.82–1.21
50 to <150	64	338	0.81	0.61–1.09
< 50	28	89	1.44	0.90–2.32

*ALL* acute lymphoblastic leukaemia, *AML* acute myeloid leukaemia, *CI* confidence interval, *OR* odds ratio *SES* socioeconomic status.

Analyses were conducted using a random intercept logistic regression model adjusted for age, sex and SES.

^a^Some controls overlap for ALL and AML analyses

### Confounder analyses

Table S3 provides results for the association of potential confounders with childhood leukaemia risk, adjusted for age, sex and SES. Most potential confounders examined, including traffic, urban vs. rural setting and SES, were not associated with risk of childhood leukaemia. Calculated MFs ≥ 0.4 μT were also not related to childhood leukaemia (OR: 1.07; 95% CI: 0.65–1.76) in these studies. An association between mobility and leukaemia risk was observed; the odds of leukaemia among participants who had ever moved between birth and diagnosis was 1.89 times higher than among those who had never moved (95% CI: 1.50–2.38). Participants living in single-family homes had lower odds of leukaemia than those living in other types of residences (OR: 0.80; 95% CI 0.61–1.06), but results were imprecise.

Table 5 presents ORs for the association between distance from power lines and childhood leukaemia risk with and without adjusting for specific potential confounders. Different subsets of studies are included in each analysis due to the availability of variables in the studies. The association between power lines and childhood leukaemia was slightly higher among the studies that included individual measures of SES compared to those with ecological SES measures, but adjusting for SES did not change the observed risk estimates in either subset (Table 5). Adjustments for other confounders, including dwelling type, traffic and urban vs. rural setting, also had little impact on the risk estimates. Adjustment for mobility, which was associated with leukaemia risk (Table S3), did not affect the risk estimates either (Table 5). Further investigation determined that only two studies, Brazil and Sweden, contributed meaningfully to estimating the OR in this model, and mobility was associated with distance < 50 m positively in Brazil and negatively in Sweden, which resulted in an overall lack of association. Adjusting for MF exposure using calculated fields did not materially change the OR for distance < 50 m. Including Brazil, the only measurement-based study, in these analyses strengthened the association between proximity to power lines and childhood leukaemia from 1.32 to 1.47 (95% CI: 0.83–2.60) when adjusting for MF (Table 5), but results were imprecise.

**Table 5:** Comparison of the odds ratios for association between childhood leukaemia and distance to closest overhead 200 + kV power line with and without adjustment for specific confounders

Confounder model	≥ 300 m	150 to < 300 m	50 to < 150 m	< 50 m
Ecological SES—studies 2, 3, 8, 9, 10, 11				
Not adjusted^a^	1.00 (reference)	1.01 (0.87–1.18)	0.90 (0.72–1.12)	1.28 (0.85–1.93)
Adjusted^b^	1.00 (reference)	1.02 (0.87–1.18)	0.90 (0.72–1.12)	1.28 (0.85–1.93)
Individual SES—studies 1, 5, 6, 7, 11				
Not adjusted^a^	1.00 (reference)	0.83 (0.63–1.10)	1.09 (0.77–1.54)	1.49 (0.85–2.59)
Adjusted^b^	1.00 (reference)	0.83 (0.63–1.10)	1.09 (0.77–1.55)	1.48 (0.85–2.58)
Mobility—studies 1, 5, 6, 7, 9				
Not adjusted^a^	1.00 (reference)	0.90 (0.43–1.90)	1.84 (1.00–3.38)	2.05 (0.78–5.36)
Adjusted^b^	1.00 (reference)	0.87 (0.41–1.86)	1.72 (0.93–3.20)	2.09 (0.79–5.51)
Dwelling type—studies 1, 6, 7, 11				
Not adjusted^a^	1.00 (reference)	0.95 (0.51–1.79)	1.64 (1.04–2.58)	2.59 (1.35–4.99)
Adjusted^b^	1.00 (reference)	0.96 (0.51–1.81)	1.66 (1.05–2.61)	2.62 (1.36–5.03)
Traffic—studies 3, 4, 7, 8				
Not adjusted^a^	1.00 (reference)	0.99 (0.77–1.26)	1.02 (0.73–1.42)	1.78 (1.06–2.98)
Adjusted^b^	1.00 (reference)	0.98 (0.77–1.26)	1.01 (0.72–1.41)	1.77 (1.05–2.97)
Urban setting—studies 1, 2, 3, 6, 7, 8, 10				
Not adjusted^a^	1.00 (reference)	1.02 (0.87–1.21)	1.01 (0.80–1.28)	1.28 (0.81–2.02)
Adjusted^b^	1.00 (reference)	1.02 (0.87–1.21)	1.01 (0.80–1.28)	1.28 (0.81–2.02)
Calculated fields—studies 2, 4, 5, 6, 7, 10, 11				
Not adjusted^a^	1.00 (reference)	0.95 (0.79–1.13)	0.98 (0.75–1.26)	1.16 (0.71–1.91)
Adjusted^b^	1.00 (reference)	0.95 (0.79–1.13)	1.00 (0.75–1.32)	1.23 (0.67–2.26)
Measured or calculated fields—studies 1, 2, 4, 5, 6, 7, 10, 11				
Not adjusted^a^	1.00 (reference)	0.95 (0.79–1.14)	0.97 (0.75–1.24)	1.32 (0.81–2.13)
Adjusted^b^	1.00 (reference)	0.95 (0.79–1.14)	0.98 (0.75–1.28)	1.47 (0.83–2.60)

*SES* socioeconomic status

Studies: 1, Brazil; 2, Denmark; 3, France; 4, Italy1; 5, Italy2; 6, Norway; 7, Sweden; 8, Switzerland; 9, Tasmania; 10, United Kingdom; 11, United States.

^a^Analyses were conducted using a random intercept logistic regression model, adjusting for age, sex and SES (except in SES models) in subjects who did not have missing values for the covariate of interest.

^b^Analyses were conducted using a random intercept logistic regression model, adjusting for age, sex, SES and the covariate of interest

Analyses of the association between distance and leukaemia risk stratified by various covariates revealed stronger associations with distance for participants who had ever moved and for participants from both single-family homes and other dwelling types, suggesting potential interaction effects between these covariates and proximity to power lines (Table S4). However, some results were based on small numbers and the OR for distance among participants who had ever moved was driven by a single study (Sweden). In analysis stratified by MF level, there were too few observations in the category ( < 0.1 μT and < 50 m to 200 + kV line); therefore, we used a cut point of < 0.2 μT and collapsed eight age categories to three, to achieve meaningful comparisons. A raised OR was observed in the ≥ 0.4 μT stratum, for the < 50 m to 200 + kV line category (OR: 6.25; 95% CI: 0.94–41.52), but based on small numbers.

### Sensitivity analyses

The association between distance (< 50 m compared with ≥ 300 m) to 200 + kV power lines and childhood leukaemia was stronger for diagnosis homes (OR: 1.78; 95% CI: 1.13–2.81) compared with birth homes (OR: 1.23; 95% CI: 0.79–1.91), although the CIs overlap (Table S5). This was true even in the subset of studies that had information on both birth and diagnosis homes (Table S6). When using all available data, including observations with less accurate geocoding, the minimally adjusted model provided an OR of 1.33 (95% CI: 0.92–1.91) for the shortest distance category to a 200 + kV power line (Table S5), similar to the observed association using only accurately geocoded observations (Table 3). In the analysis with all alternative controls, the association weakened in comparison with the one observed in the primary analysis. Results were broadly similar for controls for other cancer types (Table S5).

## Discussion

We conducted a pooled analysis assessing proximity to overhead power lines and its association with childhood leukaemia using individual-level data from 11 case–control studies. We found virtually no increase in risk of leukaemia among children who lived within any distance (including < 50 m) to power lines of all voltages combined. We found a small, but imprecise, increase in risk of leukaemia among children who lived in homes < 50 m from higher voltage (200 + kV) power lines. We found no material association between childhood leukaemia and MF in this set of studies.

We did not find any association between childhood leukaemia and urban vs. rural, type of dwelling, traffic density or SES in this set of studies. Further, adjusting for SES did not alter the associations whether ecological or individual measures of SES were used. Unfortunately, only the US study measured both types of SES; thus, we were unable to compare these measures of SES in the pooled analysis. A previous analysis of the US data^23^ found that SES, as an individual or ecological measure, was not clearly associated with the risk of childhood leukaemia or its major subtypes.

Of the potential confounders that we examined, only mobility was associated with childhood leukaemia. Brazil obtained some of their data through interviews (however, data included in our main analysis were records based) and therefore the data on mobility were prone to non-responder bias (9.5% of cases and 12% of controls refused participation). The stratified analyses showed a much stronger association between proximity to power lines and childhood leukaemia for those who moved compared with those who never moved, but both strata had small numbers in their highest exposed categories (Table S4). Given the uncertain relationship between mobility and proximity to power lines, the support for mobility as a confounder appears limited.

We found higher ORs for distance when only studies with information on mobility, type of dwelling or traffic were included; however, adjustments for these confounders had no effect on the estimates. Thus, these variables did not appear to confound the associations, but rather indicated potential selection of studies with higher ORs for close distance, perhaps due to higher quality of studies with more detailed examination of potential confounders and more accurate geocoding.

Nevertheless, the role of mobility in the studies of childhood leukaemia is not fully understood. Assessment of that role is complicated, because it might be related to the age of the child, SES, type of housing (single-family vs. apartments), likelihood of successful geocoding, inclusion into the measurement component of the study or exposure misclassification. Further exploration of the role of mobility on the association between proximity to power lines and childhood leukaemia is warranted, whether it is through selection bias, confounding or measurement error or as a risk factor itself.

In the age-stratified analyses, excess leukaemia risk associated with close distance to power lines was limited to the younger age group, for whom any address might be more indicative of lifetime exposure and/or exposure during a critical time period. On the other hand, although we might expect exposure in birth homes to be more representative of exposure during the critical developmental time period, power line proximity to diagnosis homes was more strongly associated with childhood leukaemia than proximity to birth homes. This was the case when all studies were considered and when limiting to studies that had information on both birth and diagnosis homes (Table S6). Another possible explanation for variation with age is the heterogeneity of childhood leukaemia, involving a spectrum of lymphoid and myeloid diseases with different distributions of age at diagnosis and potentially differing aetiologies.

We did not confirm a sharp monotonic decline in the association in more recent decades as was suggested by a UK study^12^ with some support from the Danish study^13^. When the UK data were excluded, the associations by period of diagnosis were similar (Table S2). We used tighter distance intervals compared with the UK study closest distance of < 200 m, which spans three of our distance categories. Studies in our pooled analysis had little overlap across time periods and mostly smaller studies contributed cases before 1990 with the non-UK studies in total contributing roughly equal numbers of highly exposed subjects as the UK study in this period. Thus, although we did not confirm the UK finding, excluding the United Kingdom, there is only a slight suggestion of higher risk in the earliest period; all estimates are too imprecise to draw firm conclusions either way. Due to small numbers, it is difficult to explore this further even in this pooled analysis.

Similarly, other methodologic considerations fail to offer good explanations for the observed association in our study. We only included record-based studies to reduce the possibility of selection bias in our results^61^. Some studies identified subjects with poor geocoding accuracy and excluded them from analysis. Exposure misclassification due to measurement error and potential selection bias was likely minimal, as the risk estimate did not change when including less accurately geocoded observations, although very little of the poor geocoding occurred at close distances. Similarly, and as expected, the use of alternative controls reduced the risk estimates somewhat, but did not suggest strong bias. Once again, this observation may be due to the selection of the set of studies.

In addition to increasing statistical power, IPD meta-analyses (or pooling) allowed us to standardise inclusion criteria and analyses across studies, and conduct analyses that were not done or possible in the individual studies^60^. Increasing the precision of the estimates is especially important if the possible effect estimate is small, such as the association between proximity to power lines and childhood leukaemia. Pooling also strengthened the study with standardisation of data across studies, as the definitions of outcome, exposure and potential confounders varied substantially between individual studies. Particularly problematic were varied definitions of ‘exposed’ and reference categories for distance to power lines used in previous studies of childhood leukaemia. Further, pooled analysis enabled consistent application of statistical analyses to all included studies, minimising bias and resulting in more stable results.

There are inherent limitations when pooling data. First, the pooled dataset is only as good as the underlying data. Second, each study collected different information, which limited the adjustment and confounder analysis or required excluding studies. Restrictions to smaller subsets of studies in the sensitivity analyses are likely selective and not generalisable to the broader set of data.

Although the studies we have included do not show an association with MF, our results are broadly consistent with previous pooled analyses of MF and childhood leukaemia^2–5^ in that the elevated risk we found was limited to < 50 m of a 200 + kV lines, a distance at which MF are more likely to be elevated. On the other hand, the lack of association with MF and the fact that adjusting for MF did not weaken the association for distance supports alternative explanations for the associations observed between residential distance from power lines and leukaemia risk, such as other correlates of distance or unmeasured confounders. Furthermore, although we included only record-based studies, which are less prone to bias, our results are somewhat weaker and less precise than that of previous MF pooled analyses, again arguing against MF as an explanation.

In conclusion, we found a small, imprecise association between childhood leukaemia and residence located within 50 m of 200 + kV lines, which was stronger for younger children, in our individual-data pooled analysis of 11 studies. This association was not explained by exposure to high MF levels or by other measured confounders. We found no evidence for bias as a potential explanation and in particular, we only included record-based studies, making selection bias unlikely. Although exposure misclassification is likely to be present, the risk of bias due to distance misclassification is quite small. The previous UK findings of risk estimates for distances beyond 200 m are not supported by the pooled data from other countries. The decrease in effect over time are not clearly supported by the pooled data from other countries, although numbers of exposed cases and controls for the earlier time period are small for both the United Kingdom and for other countries combined. Although pooled analysis is a powerful approach to integrating data, it is only as good as the underlying data. Reasons for the small yet fairly consistent increase in the risk of childhood leukaemia in relation to proximity to power lines found in many studies remain to be elucidated.

## Electronic supplementary material

Table S1

Table S2

Table S3

Table S4

Table S5

Table S6

Figure S1

Figure S2

## Availability of data and materials

The data that supports the findings of this study are available from individual study centres, but restrictions apply to the availability of these data, which were used under license for the current study, and so are not publicly available. Data are, however, available from the authors upon reasonable request and with permission of local ethics committees and/or other constraints.
